# Antimicrobial Stewardship Programs in Northwest China: A Cross-Sectional Survey of Perceptions, Involvement, and Perceived Barriers Among Hospital Pharmacists

**DOI:** 10.3389/fphar.2021.616503

**Published:** 2021-04-29

**Authors:** Wenjing Ji, Khezar Hayat, Dan Ye, David J. McIver, Kangkang Yan, Muhtar Kadirhaz, Li Shi, Xiaofeng Liu, Hanjie Chen, Yu Fang

**Affiliations:** ^1^ Department of Pharmacy Administration and Clinical Pharmacy, School of Pharmacy, Xi’an Jiaotong University, Xi’an, China; ^2^ Center for Drug Safety and Policy Research, Xi’an Jiaotong University, Xi’an, China; ^3^ Institute of Pharmaceutical Sciences, University of Veterinary and Animal Sciences, Lahore, Pakistan; ^4^ Department of Pharmacy, Xi’an No .3 Hospital, The Affiliated Hospital of Northwest University, Xi’an, China; ^5^ Global Health Group, Institute for Global Health Sciences, University of California, San Francisco, CA, United States; ^6^ Department of Pharmacy, People’s Hospital of Ningxia Hui Autonomous Region, Yinchuan, China; ^7^ Department of Pharmacy, Qinghai Provincial People’s Hospital, Xining, China

**Keywords:** antimicrobial stewardship program, antimicrobial resistance, hospital pharmacist, participation, barriers, strategy

## Abstract

**Background:** Antimicrobial stewardship (AMS) is a key prevention strategy in addressing the global concern of increasing antimicrobial resistance (AMR). Pharmacists are one of the integral members of AMS hospital teams around the world. Toward reducing AMR, a major strategy in China is to improve the capacity and participation of pharmacists in the AMS framework. However, little is known about how hospital pharmacists perceive their position and participation in AMS work, and the barriers to this work in China, especially in the Northwest region.

**Methods:** Region this work describes a cross-sectional, anonymous, online survey study. Hospital pharmacists from five provinces/autonomous regions in northwest China were invited to participate in June and July 2020. Participants completed the survey by using WeChat, a popular social application in China. We purposefully distributed the questionnaire link and QR code to hospital pharmacists through the hospital antimicrobial resistance surveillance network, hospital antimicrobial consumption surveillance network, provincial and city pharmaceutical associations, and hospital pharmacist WeChat groups.

**Results:** Out of 1032 respondents, 93.1% believed that AMS programs promote the judicial prescribing of antimicrobials, 95.5% strongly agreed that AMS could reduce the widespread use of antimicrobials, and 92.3% believed that AMS could improve medical services. Pharmacists were most likely to be involved in AMS through reviewing prescriptions of antimicrobials, intervening in inappropriate prescriptions, and providing feedback on antimicrobial prescriptions and medical orders. Barriers to participating in AMS included workload (59.5% of respondents), ineffective communication between pharmacists and doctors (57.7%), and inadequate knowledge of AMS (47.0%). Differences in responses were found between the five surveyed provinces. A significant association was found between median involvement scores and gender, age, education, level of superiority, experience, and type of hospital (*p* < 0.05).

**Conclusion:** Pharmacists perceived that AMS programs are important, but that their involvement in related activities is limited in all provinces. Further studies and strategies should consider how to overcome the identified barriers to optimize the participation of pharmacists in AMS programs.

## Introduction

Antimicrobial resistance (AMR) is a growing global threat and a serious public health crisis in countries worldwide (World Health Organization, 2019). It was estimated that by 2050, 10 million lives a year and a cumulative USD 100 trillion of economic output are at risk due to the rise of drug-resistant infections if AMR is not adequately controlled (O’Neill, 2016). New AMR mechanisms are emerging and spreading globally, while the discovery of new antimicrobials remains a challenge. Antimicrobial overuse and misuse are recognized as the main drivers for the development of resistance (O’Neill, 2016). Optimizing the use of antimicrobials is critical to treat infections and combat AMR effectively.

Antimicrobial stewardship (AMS) is considered an imperative approach for optimizing the use of antimicrobial drugs, especially in clinical settings (Davey et al., 2017). Many studies have assessed the impact of AMS programs in hospitals and found that they can increase infection cure rates, improve clinical outcomes, reduce AMR and health-care-associated infections, and reduce health-care costs (Schuts et al., 2016; Huebner et al., 2019; Belan et al., 2020). In 2011, China established a comprehensive management system and technical support framework for AMS, including guidelines, regulations, surveillance networks, and relevant training sessions (Xiao, 2018). After a national AMS Campaign launched in China from 2011 to 2016, antibiotic consumption and inappropriate drug use in secondary and tertiary hospitals were successfully reduced (Xiao et al., 2020). The proportion of outpatients and surgical patients who received antibiotic treatment decreased from 19.5 to 8.5% and from 97.9 to 38.3%, respectively. Moreover, the intensity of antibiotic use among inpatients decreased from 85.3 to 48.5%, as defined daily dosage (DDD) per 100 patient days (Xiao et al., 2020).

Pharmacists, particularly those with specific training and experience in antibiotic stewardship, are key members of AMS hospital teams worldwide. Highly effective hospital AMS programs have been found to have strong pharmacist engagement, either as a leader or co-leader of the program (Heil et al., 2016). The National Health and Family Planning Commission (NHFPC) has issued several regulatory documents regarding promoting the rational use of an antimicrobial agents and containing resistance in China since 2002. The NHFPC has emphasized that AMS programs require a team of highly professional and cooperative personnel, including pharmacists. According to the requirements of the Chinese Ministry of Health (MoH, formerly NHFPC), by 2011, all tertiary hospitals and more than half of the secondary hospitals already had clinical pharmacists who are engaged in patient drug therapy, therapeutic drug monitoring, and patient medication counseling (Ministry of Health, 2008). However, there is much room to improve the capacity of pharmacists and their participation in antimicrobial therapy within China. The National Action Plan to Curb Antimicrobial Resistance (2016–2020) was launched by the MoH in 2016, as the official response from the Chinese government to the global action plan of the World Health Organization (Xiao, 2016). The Notice on Continuous Doing Well in the Management of Clinical Use of Antimicrobial Drugs was issued in July 2020, which describes strategies to improve the capacity of pharmacists in antimicrobial resistance prevention and control and participation of clinical pharmacists in the management of antimicrobial usage. Furthermore, it provides regular training for pharmacists on the knowledge and standardized management of antimicrobial use.

To maximize the value of pharmacists, it is vital to understand the current status of hospital pharmacists' participation in AMS in China, to explore perceptions of pharmacist involvement in AMS, and to understand their potential barriers to participation. To our knowledge, no research has been conducted to investigate the involvement, confidence, or challenges of the hospital pharmacist in AMS in northwest China. We, therefore, aim to fill this research gap and provide scientific evidence for future policy decisions.

## Methods

### Study Design and Setting

This is a cross-sectional online survey performed between June and July 2020. The study participants were pharmacists working in hospitals of five provinces/autonomous regions in northwest China (Shaanxi, Gansu, Ningxia, Qinghai, and Xinjiang).

### Questionnaire

The questionnaire was constructed and conceptualized based on a literature review and studying the local situation in China (Weier et al., 2018). The questionnaire was translated from english into Mandarin by a language expert. Considering that hospital pharmacists are very busy in their daily work, efforts were made to make the questionnaire clear and concise. The questionnaire was validated by an expert panel of a multidisciplinary research team, including three experienced clinical pharmacists, one epidemiologist, one director of a hospital pharmacy, and two professors majoring in pharmaceutical administration. A pilot study was also conducted among a small group with 12 hospital pharmacists from a tertiary hospital in Xi’an, the capital city of Shaanxi province, in order to optimize the clarity of the language and structure of the questionnaire. Data from the pilot study were not included in the final analysis.

The final version of the questionnaire consisted of 47 items in five sections: 1) demographics, such as gender, age, province and city, degree, and number of working years, 2) pharmacist’s perception on the importance of AMS, quantified using a 5-point Likert scale (“strongly disagree,” “disagree,” “neutral,” “agree,” and “strongly agree”), 3) pharmacist’s involvement in AMS activities, with answers ranging from “never,” “seldom,” “sometimes,” “often,” and “always”. 4) pharmacist’s confidence to participate in AMS activities (“strongly unconfident,” “unconfident,” “neutral,” “confident”, and “strongly confident,”) and 5) pharmacist’s perceptions of barriers to participating in AMS activities (5-point Likert scale). The value of Cronbach’s alpha measured for sections two to five was greater than seven based on pilot data, indicating an acceptable level of internal consistency.

### Sampling

Convenience and snowball sampling techniques were used for collection of data from respondents. The Raosoft online sample size calculator was used to estimate the sample sized by considering a 5% margin of error, 99% confidence interval and 50% response distribution. The calculated estimated sample size for this study was 653, however the actual sample size was higher.

### Data Collection

An internet survey tool, WenJuanXing web-application (https://www.wjx.cn/), was used to create an electronic questionnaire to collect data. Participants completed the survey using WeChat (Tencent, China), a popular instant messaging and social networking application. We disseminated the questionnaire link and QR code to hospital pharmacists through the following WeChat groups: the hospital antimicrobial resistance surveillance network, hospital antimicrobial consumption surveillance network, the provincial and city pharmaceutical associations, the head/director of the pharmacy, and clinical pharmacist group. All the above WeChat groups are the most important platforms for communication and information transfer among pharmacists. These WeChat groups, therefore, covered most of the pharmacists in each province, ensuring a representative sample of the survey participants. We also asked participants, in particular, the head/director of the pharmacy facility, to forward the survey link or QR code to their department and other potentially interested pharmacists. Participants were informed that the survey was anonymous and voluntary.

### Statistical Analysis

All respondents were included in the analyses. Descriptive statistical analyses were presented as frequencies (percentages) or median (with Inter-Quartile Range, IQR). For items with a 5-point Likert scale, measures were recoded as “1” for “strongly agree” or “agree,” “0” for “neutral” or “disagree” or “strongly disagree.” Median scores of AMS items were calculated and compared using demographic variables. Chi-square tests were used to compare categorical variables. Considering the scores showed skewed distribution, the significance of the scores were assessed by nonparametric tests. The Mann-Whitney test for comparing two independent groups and the Kruskal-Wallis for comparing more than two groups were used. Differences were considered statistically significant if *p* < 0.05. Analyses were performed using SAS version 9.4 (SAS Institute).

### Ethics

The study was approved by the Ethics Committee for Medical Research of Xi’an Jiaotong University on April 2, 2020 (No. 2020-1304). Before completing the questionnaire, participants were informed that data would be collected anonymously, and all data would be used for scientific purpose only.

## Results

### Demographics

Of the 1032 respondents who participated in this survey, 71.3% were female, 75.5% were 30–50 years of age, 65.5% had a bachelor's degree, and 31.8% had 6–10 years of work experience. Most of the respondents were from Shaanxi province (40.9%), followed by Xinjiang (21.4%) and Ningxia (12.3%). A large number of participants (86.7%) had undergone anti-infection related training. Table 1 shows detailed information on the characteristics of hospitals and respondents.

**TABLE 1:** Characteristics of participating hospital pharmacists.

Characteristics	Total	By study province				
		Shaanxi	Gansu	Ningxia	Qinghai	Xinjiang
	(*n* = 1032)	(*n* = 423)	(*n* = 98)	(*n* = 221)	(*n* = 70)	(*n* = 220)
	*n* (%)	*n* (%)	*n* (%)	*n* (%)	*n* (%)	*n* (%)
Pharmacist characteristics						
Gender						
Male	296 (28.7)	106 (25.1)	32 (32.7)	46 (20.8)	28 (40.0)	84 (38.2)
Female	736 (71.3)	317 (74.9)	66 (67.3)	175 (79.2)	42 (60.0)	136 (61.8)
Age (years)						
<30	149 (14.4)	55 (13.0)	15 (15.3)	37 (16.7)	8 (11.4)	34 (15.5)
30–50	779 (75.5)	334 (79.0)	79 (80.6)	157 (71.1)	46 (65.7)	163 (74.1)
>50	104 (10.1)	34 (8.0)	4 (4.1)	27 (12.2)	16 (22.9)	23 (10.4)
Degree						
Junior college and below	171 (16.6)	52 (12.3)	18 (18.4)	54 (24.4)	21 (30.0)	26 (11.8)
Bachelor	676 (65.5)	262 (61.9)	63 (64.3)	151 (68.3)	48 (68.6)	152 (69.1)
Masters or above	185 (17.9)	109 (25.8)	17 (17.3)	16 (7.3)	1 (1.4)	42 (19.1)
Title						
Junior	408 (39.5)	176 (41.6)	37 (37.8)	111 (50.2)	13 (18.6)	71 (32.3)
Intermediate	385 (37.3)	159 (37.6)	40 (40.8)	71 (32.1)	26 (37.1)	89 (40.4)
Deputy senior	175 (17.0)	65 (15.4)	17 (17.3)	26 (11.8)	25 (35.7)	42 (19.1)
Senior	64 (6.2)	23 (5.4)	4 (4.1)	13 (5.9)	6 (8.6)	18 (8.2)
Experience as a pharmacist (year)						
<1	4 (0.4)	3 (0.7)	0 (0.0)	1 (0.5)	0 (0.0)	0 (0.0)
1–5	172 (16.6)	75 (17.7)	16 (16.3)	36 (16.3)	8 (11.4)	37 (16.9)
6–10	328 (31.8)	145 (34.3)	38 (38.8)	74 (33.5)	10 (14.3)	61 (27.7)
11–20	257 (24.9)	107 (25.3)	24 (24.5)	54 (24.4)	11 (15.7)	61 (27.7)
>20	271 (26.3)	93 (22.0)	20 (20.4)	56 (25.3)	41 (58.6)	61 (27.7)
Level of anti-infection related training (multiple)						
None	137 (13.3)	62 (14.7)	8 (8.2)	39 (17.6)	8 (11.4)	20 (9.1)
National	259 (25.1)	100 (23.6)	31 (31.6)	29 (13.1)	29 (41.4)	70 (31.8)
Provincial	403 (39.1)	131 (31.0)	60 (61.2)	64 (28.9)	55 (78.6)	93 (42.3)
City	333 (32.3)	154 (36.4)	39 (39.8)	67 (30.3)	20 (28.6)	53 (24.1)
Hospital	567 (54.9)	225 (53.2)	51 (52.0)	116 (52.5)	36 (51.4)	139 (63.2)
Pharmaceutical society	436 (42.3)	187 (44.2)	49 (50.0)	85 (38.5)	28 (40.0)	87 (39.5)
Anti-infection society	213 (20.6)	89 (21.0)	25 (25.5)	44 (19.9)	16 (22.9)	39 (17.7)
Other	161 (15.6)	73 (17.3)	15 (15.3)	35 (15.8)	8 (11.4)	30 (13.6)
Hospital characteristics						
Hospital type						
Comprehensive	916 (88.8)	389 (92.0)	86 (87.8)	195 (88.2)	56 (80.0)	190 (86.4)
Specialty	116 (11.2)	34 (8.0)	12 (12.2)	26 (11.8)	14 (20.0)	30 (13.6)
Hospital type						
Public	989 (95.8)	391 (92.4)	96 (98.0)	217 (98.2)	66 (94.3)	219 (99.5)
Private	43 (4.2)	32 (7.6)	2 (2.0)	4 (1.8)	4 (5.7)	1 (0.5)

### Importance of Antimicrobial Stewardship Programs.

Most pharmacists perceived AMS as important in fulfilling multiple objectives (Supplementary Figure S1). A large proportion of hospital pharmacists (*n* = 961, 93.1%) believed that AMS programs promote the reasonable prescribing of antimicrobials, which will help to control the risk of antimicrobial resistance. A similar proportion (*n* = 985, 95.5%) agreed or strongly agreed with the statement that “AMS could reduce the overall use of antimicrobials.” Likewise, 92.3% (*n* = 952) of the respondents believed that AMS could improve medical services, and 88.7% (*n* = 915) thought that AMS could reduce the cost of treatment. There were no significant differences between the five provinces in the perceived importance of AMS (see Table 2).

**TABLE 2:** Pharmacist’s perception toward the importance of AMS (combined agree or strongly agree response).

Items	Total	Shaanxi	Gansu	Ningxia	Qinghai	Xinjiang	*p-*value
	(*n* = 1032)	(*n* = 423)	(*n* = 98)	(*n* = 221)	(*n* = 70)	(*n* = 220)	
	*n* (%)	*n* (%)	*n* (%)	*n* (%)	*n* (%)	*n* (%)	
Promotes reasonable prescription of antimicrobial and helps control bacterial resistance	961 (93.1)	395 (93.4)	94 (95.9)	200 (90.5)	65 (92.9)	207 (94.1)	0.413
Reduces the overuse of antimicrobials	985 (95.5)	404 (95.5)	96 (98.0)	204 (92.3)	68 (97.1)	213 (96.8)	0.097
Reduces the cost of treatment	915 (88.7)	370 (87.5)	88 (89.8)	191 (86.4)	61 (87.1)	205 (93.2)	0.168
Improves medical quality	952 (92.3)	388 (91.7)	93 (94.9)	196 (88.7)	67 (95.7)	208 (94.6)	0.098

### Involvement in Antimicrobial Stewardship Programs

As outlined in Table 3 and Supplementary Figure S2, more than half of the respondents were often or always involved in reviewing the prescription of antimicrobials (*n* = 628, 60.9%) and proactively intervened in inappropriate prescriptions of antimicrobials (*n* = 544, 52.7%). Likewise, 51.3% of respondents often or always provided feedback on antimicrobial prescriptions and medical orders. However, fewer respondents said that they often or always participate in the decision-making of antimicrobial treatment for critically ill patients (*n* = 176, 17.1%) or at AMS committee meetings (*n* = 214, 20.7%). About a quarter of the respondents said that they often or always provide advice to doctors for the conversion of intravenous to oral antimicrobial drugs (*n* = 251, 24.3%), including the side effects of antimicrobials (*n* = 303, 29.4%). Only a limited number of respondents took part in monitoring the duration of antimicrobial administration (*n* = 223, 21.6%). Similarly, the number of hospital pharmacists who often or always communicated with doctors (*n* = 254, 24.6%), patients, and family members about antimicrobial treatment was low (*n* = 127, 12.3%). Overall, the proportion of pharmacist involvement in AMS activity in Qinghai province was significantly higher than in others. The median involvement score of hospital pharmacists toward AMS was significantly associated with gender, age, education, level of seniority, experience, and type of hospital (*p* < 0.05). The pharmacists who were male, with higher education, higher position, and more experience, were much more involved in AMS activities than others (Table 4).

**TABLE 3:** Involvement of the pharmacist in AMS activity (combined often or always response).

Items	Total	Shaanxi	Gansu	Ningxia	Qinghai	Xinjiang	*p-*value
	(*n* = 1032)	(*n* = 423)	(*n* = 98)	(*n* = 221)	(*n* = 70)	(*n* = 220)	
	*n* (%)	*n* (%)	*n* (%)	*n* (%)	*n* (%)	*n* (%)	
Review prescription of antimicrobials	628 (60.9)	235 (55.6)	66 (67.4)	131 (59.3)	54 (77.1)	142 (64.6)	0.003
Proactively intervene in problematic prescriptions of antimicrobials	544 (52.7)	196 (46.3)	49 (50.0)	120 (54.3)	49 (70.0)	130 (59.1)	0.001
Comments on antimicrobial prescriptions and medical orders	521 (50.5)	189 (44.7)	58 (59.2)	101 (45.7)	52 (74.3)	121 (55.0)	<0.001
Provide feedback on antimicrobial prescriptions and medical orders	529 (51.3)	189 (44.7)	52 (53.1)	106 (48.0)	56 (80.0)	126 (57.3)	<0.001
Participate in decision-making of antimicrobial treatment for critically ill patients	176 (17.1)	68 (16.1)	13 (13.3)	34 (15.4)	17 (24.3)	44 (20.0)	0.227
Participate in AMS committee meetings and decision making	214 (20.7)	79 (18.7)	16 (16.3)	46 (20.8)	31 (44.3)	42 (19.1)	<0.001
Provide advice to doctors regarding the intravenous-to-oral conversion of antimicrobials	251 (24.3)	90 (21.3)	23 (23.5)	57 (25.8)	21 (30.0)	60 (27.3)	0.320
Provide advice to doctors about the adverse drug effects of antimicrobials	303 (29.4)	105 (24.8)	31 (31.6)	66 (29.9)	30 (42.9)	71 (32.3)	0.021
Provide advice to doctors about drug interactions related to antimicrobials	269 (26.1)	93 (22.0)	24 (24.5)	59 (26.7)	28 (40.0)	65 (29.6)	0.016
Monitor the duration of antimicrobial treatment and recommend appropriate cessation	223 (21.6)	75 (17.7)	20 (20.4)	49 (22.2)	23 (32.9)	56 (25.5)	0.026
Participate in developing hospital antimicrobial treatment guidelines	220 (21.3)	91 (21.5)	21 (21.4)	39 (17.7)	31 (44.3)	38 (17.3)	<0.001
Provide feedback to doctors on bacterial resistance	242 (23.5)	89 (21.0)	18 (18.4)	49 (22.2)	29 (41.4)	57 (25.9)	0.003
Participate in the reminder of antimicrobial use in the hospital information system	249 (24.1)	85 (20.1)	25 (25.5)	50 (22.6)	33 (47.1)	56 (25.5)	<0.001
Participate in the management and evaluation of antimicrobial types in hospital	252 (24.4)	90 (21.3)	21 (21.4)	52 (23.5)	38 (54.3)	51 (23.2)	<0.001
Monitor and evaluate antimicrobial consumption in hospital	271 (26.3)	103 (24.4)	22 (22.5)	45 (20.4)	41 (58.6)	60 (27.3)	<0.001
Communicate with doctors on anti-infective treatment	254 (24.6)	94 (22.2)	24 (24.5)	47 (21.3)	29 (41.4)	60 (27.3)	0.007
Communicate with patients and family members on antimicrobial treatment	127 (12.3)	39 (9.2)	13 (13.3)	37 (16.7)	9 (12.9)	29 (13.2)	0.091

**TABLE 4:** Median scores of pharmacists rating their involvement and perception in AMS activities based on demographics.

Characteristics	Importance^a^		Involvement		Confidence		Barriers	
	Median (IQR)	*p-*value	Median (IQR)	*p-*value	Median (IQR)	*p-*value	Median (IQR)	*p-*value
Gender								
Male	4.50 (4.00–5.00)	0.574	2.94 (2.00–3.71)	<0.001	3.57 (3.00–4.00)	<0.001	3.29 (0.68)	0.326
Female	4.50 (4.00–5.00)		2.41 (1.76–3.24)		3.14 (2.86–3.86)		3.24 (0.65)	
Age (years)								
<30	4.25 (4.00–5.00)	0.105	2.00 (1.59–2.88)	<0.001	3.00 (2.86–3.57)	<0.001	3.26 (0.66)	0.055
30–50	4.50 (4.00–5.00)		2.58 (1.82–3.35)		3.14 (2.86–3.86)		3.27 (0.67)	
>50	4.75 (4.00–5.00)		3.24 (2.53–3.88)		3.86 (3.29–4.00)		3.12 (0.54)	
Degree								
Junior college and below	4.00 (4.00–5.00)	<0.001	2.47 (1.76–3.24)	<0.001	3.29 (3.00–4.00)	<0.001	3.33 (0.72)	0.003
Bachelor	4.50 (4.00–5.00)		2.47 (1.76–3.29)		3.14 (2.86–3.86)		3.27 (0.63)	
Masters or above	4.75 (4.00–5.00)		2.94 (2.12–3.53)		3.71 (3.00–4.00)		3.11(0.66)	
Title								
Junior	4.25 (4.00–5.00)	<0.001	2.09 (1.54–2.88)	<0.001	3.00 (2.86–3.57)	<0.001	3.31 (0.66)	<0.001
Intermediate	4.50 (4.00–5.00)		2.53 (1.82–3.29)		3.29 (2.71–4.00)		3.27 (0.68)	
Deputy senior	4.75 (4.00–5.00)		3.29 (2.59–3.82)		3.71 (3.00–4.00)		3.19 (0.58)	
Senior	4.63 (4.00–5.00)		3.59 (3.06–3.88)		4.00 (3.71–4.00)		2.95 (0.57)	
Experience as a pharmacist (years)								
<1	4.50 (3.75–4.50)	0.123	1.85 (1.57–3.01)	<0.001	4.00 (2.93–4.43)	<0.001	2.92 (0.42)	0.094
2–5	4.25 (4.00–5.00)		2.24 (1.71–2.94)		3.14 (2.86–3.71)		3.24 (0.68)	
6–10	4.50 (4.00–5.00)		2.29 (1.71–3.18)		3.00 (2.71–3.86)		3.30 (0.64)	
11–20	4.25 (4.00–5.00)		2.47 (1.76–3.35)		3.29 (2.86–4.00)		3.30 (0.72)	
>20	4.50 (4.00–5.00)		3.12 (2.35–3.71)		3.57 (3.00–4.00)		3.17 (0.58)	
Hospital type								
Comprehensive	4.50 (4.00–5.00)	0.716	2.50 (1.76–3.34)	0.001	3.29 (2.86–3.86)	0.031	3.26 (0.64)	0.547
Specialty	4.25 (4.00–5.00)		3.00 (2.18–3.53)		3.43 (3.00–4.00)		3.18 (0.75)	
Hospital type								
Public	4.50 (4.00–5.00)	0.144	2.59 (1.79–3.35)	0.996	3.29 (2.86–3.93)	0.587	3.25 (0.66)	0.312
Private	4.25 (4.00–5.00)		2.59 (1.76–3.35)		3.14 (2.57–3.86)		3.35 (0.60)	

a Including all items related to the pharmacist’s perception toward the importance of AMS. The same is true for involvement, perceived, and barriers. The median score was based on a Likert scale (0 = strongly disagree and 5 = strongly agree, or 0 = never and 5 = always; or 0 = strongly un-confidence and 5 = strongly confidence).

### Confidence to Perform Antimicrobial Stewardship Activities

Approximately half of the respondents reported that they are confident in performing various AMS activities, including the review of the appropriateness of antimicrobial prescription (*n* = 506, 49.0%), determination of specific antimicrobials to manage common infections such as pneumonia and urinary tract infections (*n* = 540, 52.3%), and adjustment of frequency/dose of antimicrobials based on the individual characteristics of the patient and type of infection (*n* = 497, 48.2%). However, the level of confidence of respondents was lower during consultations and case discussions on antimicrobial treatment (*n* = 370, 35.9%). Overall, the proportions of pharmacists with high confidence in performing AMS activities were higher in Qinghai province and Xinjiang Uygur Autonomous Region compared to other provinces, as shown in Table 5. The median confidence score was significantly associated with sex, title, degree, experience, and age (*p* < 0.05) (Table 4).

**TABLE 5:** Pharmacist’s confidence in performing AMS activities (with confident or strongly confident response).

Items	Total	Shaanxi	Gansu	Ningxia	Qinghai	Xinjiang	*p-*value
	(*n* = 1032)	(*n* = 423)	(*n* = 98)	(*n* = 221)	(*n* = 70)	(*n* = 220)	
	*n* (%)	*n* (%)	*n* (%)	*n* (%)	*n* (%)	*n* (%)	
Review the appropriateness of antimicrobials use	506 (49.0)	190 (44.9)	51 (52.0)	100 (45.3)	40 (57.1)	125 (56.8)	0.020
Determine the antimicrobial type to treat common infections (such as pneumonia, urinary tract infections, etc.)	540 (52.3)	210 (49.7)	47 (48.0)	111 (50.2)	42 (60.0)	130 (59.1)	0.090
Adjust the dose and frequency of antimicrobials based on patient and infections	497 (48.2)	185 (43.7)	48 (49.0)	98 (44.3)	43 (61.4)	123 (55.9)	0.005
Optimize antimicrobial treatment based on pharmacodynamic and pharmacokinetic parameters	390 (37.8)	147 (34.8)	40 (40.8)	81 (36.7)	28 (40.0)	94 (42.7)	0.332
Provide reasonable advice on the course of antimicrobial treatment	437 (42.34)	169 (40.0)	40 (40.8)	90 (40.7)	36 (51.4)	102 (46.4)	0.273
Identify unnecessary antimicrobial treatment and suggest stopping treatment	445 (43.1)	167 (39.5)	45 (45.9)	93 (42.1)	37 (52.9)	103 (46.8)	0.157
Participate in consultation and case discussion on antimicrobial treatment	370 (35.9)	130 (30.7)	37 (37.8)	76 (34.4)	34 (48.6)	93 (42.3)	0.007

### Barriers Towards Antimicrobial Stewardship Activities

A large proportion of the respondents agreed that three major barriers are impeding the participation of hospital pharmacists in AMS activities: 1) lack of efficient communication between pharmacists and doctors (*n* = 595, 57.7%), 2) pharmacists’ limited time to participate in AMS due to other daily work (*n* = 614, 59.5%), and 3) lack of acceptance of pharmacist’s advice on antimicrobials by doctors (*n* = 565, 54.8%). As well, 47.0% (485) of the respondents also considered that their inadequate knowledge regarding antimicrobials, including their indications, dosage, duration, and adverse effects, was a barrier to participating in AMS, as highlighted in Table 6.

**TABLE 6:** Perceived barriers of the pharmacist toward participating in AMS (combined agree or strongly agree response).

Items	Total	Shaanxi	Gansu	Ningxia	Qinghai	Xinjiang	*p-*value
	(*n* = 1032)	(*n* = 423)	(*n* = 98)	(*n* = 221)	(*n* = 70)	(*n* = 220)	
	*n* (%)	*n* (%)	*n* (%)	*n* (%)	*n* (%)	*n* (%)	
Have limited knowledge of antimicrobial treatment (indications, dosage, duration, combination and adverse reactions, etc.)	485 (47.0)	207 (48.9)	46 (46.9)	105 (47.5)	31 (44.3)	96 (43.6)	0.760
Think attention from the hospital administration about AMS is not enough	382 (37.0)	162 (38.3)	50 (51.0)	84 (38.0)	24 (34.3)	62 (28.2)	0.003
Think participation in AMS will make no difference	185 (17.9)	75 (17.7)	18 (18.4)	50 (22.6)	13 (18.6)	29 (13.2)	0.151
Have limited time to participate in AMS due to other daily work	614 (59.5)	245 (57.9)	65 (66.3)	131 (59.3)	54 (77.1)	119 (54.1)	0.007
Think there is a lack of efficient communication between pharmacists and doctors	595 (57.7)	242 (57.2)	65 (66.3)	132 (59.7)	47 (67.1)	109 (49.6)	0.018
Believe the pharmacist’s advice on antimicrobials has not been widely accepted by doctors	565 (54.8)	243 (57.5)	63 (64.3)	128 (57.9)	40 (57.1)	91 (41.4)	<0.001

Only the level of seniority of pharmacists and their achieved degree was noted to have a significant association with the median barrier score (*p* < 0.05), with lower seniority and earned a degree reflecting greater perceived the barriers (Table 4).

## Discussion

Antimicrobial resistance is a public health crisis that seriously threatens patient outcomes. AMS optimizes patient outcomes while minimizing the unintended consequences of antimicrobial use. Hospital pharmacists, along with infectious disease physicians, often have the most central role in an AMS program. This study is the first to explore the perception, involvement, and perceived barriers toward AMS program involvement among Chinese hospital pharmacists. We found the participation of hospital pharmacists in AMS is limited due to several barriers, including ineffective communication between pharmacists and doctors, extensive workload, and inadequate knowledge.

Most of the participants of this study believed that AMS programs are essential for the judicial prescribing of antimicrobials, as these programs reduce the cost of care and utilization of antimicrobials. The acceptability, importance, and implementation of hospital-based AMS programs across different hospital settings is well-described in the literature due to their acknowledged benefits (Brotherton, 2018; Chen et al., 2018; Burgess et al., 2019; Onorato et al., 2020). Numerous developed countries, including the United States, United Kingdom, Australia, and Canada, have implemented AMS programs successfully (Seo et al., 2016; Charani et al., 2017; DiDiodato and McAthur, 2017; Rautemaa-Richardson et al., 2018; Cipko et al., 2020); however, its implementation in developing countries is challenging (Hayat et al., 2020). It has been proven that pharmacist-driven AMS is beneficial in reducing the duration of antimicrobial treatment, shortening hospital stays, lowering patient mortality, optimizing antimicrobials use, and limiting the emergence of multidrug-resistant organisms (Li et al., 2017; Parente and Morton, 2018; Wang et al., 2019; Sadyrbaeva-Dolgova et al., 2020; Takito et al., 2020). Therefore, the increased involvement of hospital pharmacists with AMS deserves more in-depth implementation and broader promotion.

More than half of the pharmacists in this study were involved in reviewing the prescriptions of antimicrobials, coupled with appropriate feedback. Although pharmacists should be an integral part of the stewardship team, according to our survey hospital pharmacist participation was limited to monitoring antimicrobial consumption, communicating with doctors, patients, and family members about antimicrobial treatment, developing indigenous standard treatment guidelines, and providing advice to doctors about adverse effects and drug interactions of antimicrobial agents. Thus, pharmacists are performing fewer clinical roles within hospitals than they might otherwise. This may be because the number of trained clinical pharmacists is limited in Chinese hospitals, and overburdened pharmacists cannot perform a variety of crucial clinical roles (Fang, 2016). The Chinese Ministry of Health (MoH) has issued a series of policies to ensure the availability of trained infectious disease clinical pharmacists in different hospital settings (Xiao and Li, 2013; Ministry of Health, 2014), though there has been a considerable lag time in meeting the goals of the MoH.

Various studies conducted in low- and middle-income countries (LMICs) have highlighted that pharmacists are facing difficulties in performing clinical roles owing to lack of a sufficient number of pharmacists, inadequate training, lack of incentives, poor job description, and limited accessibility of clinical files of patients (Katoue et al., 2014; Salim et al., 2016; Bilal et al., 2017; Sakeena et al., 2018). In many Chinese hospitals, clinical pharmacists are rare, as are pharmacists with infection-related backgrounds. One clinical pharmacist is usually shared by multiple departments, which reflects the lack of resources to adequately support AMS programs, including human resources. Pharmacists from Qinghai reported a higher rate of involvement than other provinces, which is probably due to the higher proportion of participants in Qinghai who have worked for more than 20 years and have higher positions.

AMS teams are comprised of different healthcare professionals, including physicians, pharmacists, and nurses, who possess unique skills and training to meet AMS goals (Mendelson et al., 2020). The literature has shown that pharmacists are an integral pillar of the AMS team (Parente and Morton, 2018; Takito et al., 2020). In our study, pharmacists said that they are unable to practice AMS activities owing to time constraints and inefficient communication with other healthcare professionals. Optimal communication between clinical pharmacists and physicians is an essential part of AMS programs, and it could further improve decision making (Curran et al., 2019). As was found in this study, a qualitative study conducted in Pakistan also reported the absence of mutual collaboration among healthcare professionals (Hayat et al., 2019a). Unfortunately, existing gaps in communication between healthcare professionals could ultimately negatively affect the quality of life of patients. Similarly, when asking the pharmacist’s confidence in participating in different activities related to AMS, the results of the current study show that only around half of the pharmacists are confident of performing dosage adjustment, prescription review, and selection of the most suitable antimicrobial agent to treat infectious diseases. Pharmacists in this study were even less confident regarding antimicrobial treatment optimization and participation in case discussions, maybe due to inadequate knowledge and training about the core elements of AMS as seen in some LMICs (Penm et al., 2014; Sakeena et al., 2018; Hayat et al., 2019a; Hayat et al., 2019b; Hayat et al., 2020). We suggest that more sustainable formal education and clinical practice should be provided for pharmacists. This will not only help them gradually improve their experience in the optimal treatment for patients, but also give them more confidence to provide education to other health professionals in hospitals, supporting their role in developing hospital guidelines on antimicrobial use.

Limited acceptance of pharmacists’ advice on antimicrobials by doctors was seen as a significant barrier by the respondents in this study, which seems to contrast with previously published studies. There are a considerable number of studies which show that doctors widely accept the clinical role of pharmacists in the provision of drug therapy (Cook and Gooch, 2015; Hale et al., 2016; Abdel-Latif, 2017; Zhang et al., 2020), and the rate of acceptance of pharmacists’ recommendations appears to be higher for patients with multiple medication orders (Zaal et al., 2020). In China although a growing number of pharmacists have been working in hospitals since 2011, and 86.7% of participants in this study said that they attended infection training, because the main task of pharmacists in Chinese hospitals has traditionally been to dispense medicines, we believe that a shortage of pharmacists with solid anti-infection training and strong clinical experience could be the main reason for the limited acceptability of their recommendations related to antimicrobials. This could also lead to the pharmacist’s feeling that their efforts are not respected. The relationship between pharmacists and doctors must be improved in order to gain mutual trust and increase pharmacist’s impact on AMS. Hospital pharmacists should devote more time toward cooperating with doctors toward improving their professional level, including but not limited to participating in ward rounds and case discussions regularly.

## Limitations

There are several limitations to this study. First, it was conducted only in five provinces located in the northwest of China. Although the overall findings were similar across provinces, the results may differ in other provinces in other parts of China. Second, this is an online survey based on WeChat groups, which might cause potential differences between the pharmacists who are in the group and those who are not. Since the WeChat groups have been the most important platform for communication between pharmacists, this was the most effective way to reach the most pharmacists. Third, this is a cross-sectional survey employing the Likert scale, and the study reported the perception and attitudes of hospital pharmacist at one point in time, and therefore it cannot capture the actual developmental processes over time. Also, intervals between points on the Likert scale might not present equal changes in attitude for all individuals. Finally, the sample size from Gansu and Qinghai provinces is small, which may show non-response bias and affect generalizability. Some pharmacists from these two provinces did not respond to requests to participate in our survey after multiple attempts to contact. Regardless of these limitations, this is the first exploratory study that provides insight into the opinions of Chinese hospital pharmacists from northwest China toward various aspects of AMS programs.

## Conclusion

We found that pharmacists from northwest China perceived that AMS programs are important; however, their participation, involvement, and confidence in AMS related activities was found to be lacking due to several barriers including limited collaboration with doctors, inadequate knowledge, and limited time. The onus is on the government to design and tailor policies to address the barriers highlighted in this study to ensure smooth operating AMS programs. Further studies should consider how to overcome the identified barriers and to optimize the participation of pharmacists in AMS. Enhancing AMS program effectiveness and administrative efficiency is a crucial step to tackling the growing problem of antimicrobial resistance.

### Recommendations

Pharmacists face many barriers to providing their true value. We propose the following strategies to assist hospital pharmacists to better participate in AMS work: 1) senior management personnel, such as hospital deans, need to be more involved in AMS activities; 2) more support for pharmacist engagement in more clinical-practice-based activities; 3) systematic education and training for pharmacists, beginning from undergraduate and graduate students majoring in pharmacy; 4) recruit more highly qualified pharmacists; 5) improve the tools and facilities necessary for AMS work, such as electronic systems; 6) create an atmosphere of interdisciplinary communication within the hospital.

## Data Availability

The data analyzed in this study is subject to the following licenses/restrictions: The datasets generated and/or analyzed during the current study are not publicly available due to privacy restrictions but are available from the corresponding author on reasonable request. Requests to access these datasets should be directed to yufang@mail.xjtu.edu.cn.
