# Supplementary material for: Antimicrobial Stewardship Programs in Northwest China: A Cross-Sectional Survey of Perceptions, Involvement, and Perceived Barriers Among Hospital Pharmacists
Source: Front Pharmacol. 2021 Apr 29;12:616503. doi: 10.3389/fphar.2021.616503 (PMC8117155; doi:10.3389/fphar.2021.616503)
Supplement: Supplementary file 1 [file datasheet1.docx]

**FIFURE 1** Pharmacist’s perception on importance of AMS in Chinese hospital

**FIGURE 2** Pharmacist’s involvement in the hospital AMS
